# Learning curve of tibial cortex transverse transport: a cumulative sum analysis

**DOI:** 10.1186/s13018-023-04149-x

**Published:** 2023-09-02

**Authors:** Jun-Peng Liu, Xing-Chen Yao, Zi-Yu Xu, Xin-Ru Du, Hui Zhao

**Affiliations:** Department of Orthopaedic Surgery, Beijing Chaoyang Hospital, Capital Medical University, Beijing, 100020 China

**Keywords:** Angiogenesis, Complication, Diabetic foot ulcer, Learning curve, Tibial cortex transverse transport, Ulcer healing

## Abstract

**Objective:**

This study aimed to describe the learning curve of surgeons performing tibial cortex transverse transport (TTT) and explore its safety and effectiveness during the initial stages of surgeon’s learning.

**Methods:**

The clinical data of patients with diabetic foot ulcers classified as Wagner grade ≥ 2, who underwent TTT at our hospital from January 2020 to July 2021, were included in this retrospective analysis. The same physician performed all procedures. Patients were numbered according to the chronological order of their surgery dates. The cumulative sum and piecewise linear regression were used to evaluate the surgeon's learning curve, identify the cut-off point, and divide the patients into learning and mastery groups. A minimum follow-up period of 3 months was ensured for all patients. Baseline data, perioperative parameters, complications, and efficacy evaluation indicators were recorded and compared between the two groups.

**Results:**

Sixty patients were included in this study based on the inclusion and exclusion criteria. After completing 20 TTT surgeries, the surgeon reached the cut-off point of the learning curve. Compared to the learning group, the mastery group demonstrated a significant reduction in the average duration of the surgical procedure (34.88 min vs. 54.20 min, *P* < 0.05) along with a notable decrease in intraoperative fluoroscopy (9.75 times vs. 16.9 times, *P* < 0.05) frequency, while no significant difference was found regarding intraoperative blood loss (*P* = 0.318). Of the patients, seven (11.7%) experienced complications, with three (15%) and four cases (10%) occurring during the learning phase and the mastery phase, respectively. The postoperative ulcer area was significantly reduced, and the overall healing rate was 94.8%. Significant improvements were observed in postoperative VAS, ABI, and WIFI classification (*P* < 0.05). There were no significant differences in the occurrence of complications or efficacy indicators between the learning and mastery groups (*P* > 0.05).

**Conclusion:**

Surgeons can master TTT after completing approximately 20 procedures. TTT is easy, secure, and highly efficient for treating foot ulcers. Furthermore, TTT’s application by surgeons can achieve almost consistent clinical outcomes in the initial implementation stages, comparable to the mastery phase.

## Introduction

In 2021, the global prevalence of type 2 diabetes mellitus (T2DM) reached approximately 537 million individuals [1]. Notably, most patients struggle to maintain their blood sugar levels within a satisfactory range. Furthermore, long-term poorly controlled blood glucose levels can trigger neuropathy affecting the feet, resulting in muscular imbalances that lead to deformities. In turn, these deformities influence weight distribution on the feet, which, combined with the absence of protective sensation, increases the risk of skin damage. Moreover, hindrance of microcirculation caused by peripheral vascular narrowing, obstruction, and microvascular alterations leads to impaired blood supply to the distal extremities [2], detrimentally affecting tissue metabolism and diminishing tissue repair and infection-fighting capabilities. The combination of these factors contributes to the development of diabetic foot ulcers (DFU), which are challenging to heal. Remarkably, the population of patients with DFU is large, affecting approximately 25% of T2DM patients [3], with amputation rates as high as 15% [4].

However, traditional treatment methods, such as surgical debridement, free-flap transplantation, infection control, and revascularization [5–9], often fail to effectively cure intractable ulcers [10]. Off-loading surgery, such as osteotomies, achieved through bone resection, can effectively rebalance plantar pressures, thereby minimizing mechanical damage and lowering the likelihood of ulcer development [11]. However, its effectiveness in addressing lower limb revascularization is limited. Research [2] has demonstrated that 90% of patients with DFU who undergo amputation have a history of foot ischemia, predominantly caused by the occlusion of small arteries [12]. Therefore, the core of DFU treatment lies in the reconstruction of distal foot circulation and the enhancement of microcirculation and oxygen metabolism in adjacent tissues. Tibial cortex transverse transport (TTT) is based on Ilizarov's law of tension stress [13], that is, appropriate tensile stress is applied to the bone to improve the expression of angiogenesis and tissue repair-related factors in the serum of patients, thereby stimulating local tissue growth, promoting the regeneration of microcirculation, and subsequently augmenting local blood supply [14]. Recently, Hua et al. [15] introduced the TTT for treating DFU, which yielded encouraging outcomes in facilitating ulcer healing.

Surgeons are eager to master this innovative technique. However, no literature currently provides a comprehensive description of the learning process and competency development of surgeons using this technique. During the initial stages of skill acquisition, the safety, efficacy, and occurrence of complications associated with TTT remain unclear. Blindly employing this procedure may lead to adverse events without appropriate guidance and standardized references. The learning curve, defined as the time or number of cases necessary for a surgeon to attain proficiency in a specific technique [16], has yet to be delineated for the TTT. The CUSUM analysis can discover initial abnormal data points in continuous variables as early as possible and scientifically evaluate the cut-off points of the learning curve [17]. Additionally, a piecewise linear model [18] can describe the learning process intuitively and with simplicity, particularly when it exhibits distinct segmentation characteristics. Hence, the purpose of this study was to analyze the learning curve of the TTT through cumulative sum (CUSUM) analysis [19] and piecewise linear regression analysis [18]. This analysis sought to determine the requisite number of cases for physicians to achieve proficiency in the TTT, assess the safety and effectiveness of this technology, and provide valuable data for its popularization.

## Methods

### Patients and study design

This retrospective analysis was conducted on patients with Wagner grade ≥ 2 DFUs who underwent TTT surgery at our hospital from January 2020 to July 2021. The study was approved by the Ethics Committee of our hospital, and informed consents were obtained from all patients.

The surgical indications were as follows: (1) patients who met the 1999 WHO diagnostic criteria for diabetes (including fasting blood glucose levels ≥ 7.0 mmol/L, postprandial 2-h blood glucose levels ≥ 11.1 mmol/L, or random blood glucose levels ≥ 11.1 mmol/L); (2) Wagner grade ≥ 2, unresponsive to over 2 months of debridement, VSD, and standard medical treatment; and (3) at least one patent arterial branch in the anterior tibial, posterior tibial, or peroneal artery without complete occlusion. Surgical contraindications were as follows: (1) inability to tolerate anesthesia or surgery; (2) recent occurrence of other severe complications of diabetes or uncontrolled infection; and (3) presence of an obvious wound or infection in the surgical incision area.

During the study period, 63 patients underwent TTT. Patients with incomplete medical histories or outcome data were excluded. As complications typically appear 3 months postoperatively [20], all patients were followed-up for 3 months after surgery in this study, and the follow-up was terminated earlier if the wound further enlarged or the patient died. The surgical team remained consistent throughout the study, and the operator had no prior experience in performing the TTT independently. To minimize subjective factors, perioperative parameters, clinical data, and complications were recorded by assessors who were not involved in surgery or daily wound care.

### Surgical technique

During the procedure, we modified the original technique [15] by reducing the osteotomy area. The surgical steps were as follows. (1) Patients were instructed to assume a supine position and routine preoperative disinfection and sterile towel spreading were performed. Most surgeries used epidural anesthesia, while a few used general anesthesia. (2) Two curved incisions, each measuring approximately 2 cm, were made 2 cm below the tibial tuberosity along the medial aspect of the tibial crest, with a gap of 3 cm between them. The tissue layers were separated until the periosteum was exposed. Two rectangular bone windows (1.5 cm × 1.5 cm) were marked on the medial side of the tibia. The periosteum was incised along the markings and carefully peeled to ensure the preservation of its integrity. (3) Two 3-mm fixation pins were inserted into each bone window (penetrating only one side of the cortical bone) to mobilize the bone segments. Holes were drilled along the edges of the bone windows using a bone drill, and the bone flaps were detached using a bone chisel to create two mobile bone flaps. (4) Two 4-mm external fixation needles were screwed into the bone (penetrating the bone cortex bilaterally). An external fixator was installed and tightened. The subcutaneous tissue and skin were meticulously sutured layer-by-layer under sterile conditions, followed by the application of a sterile dressing and bandage.

### Postoperative management

**Fig. 1:**
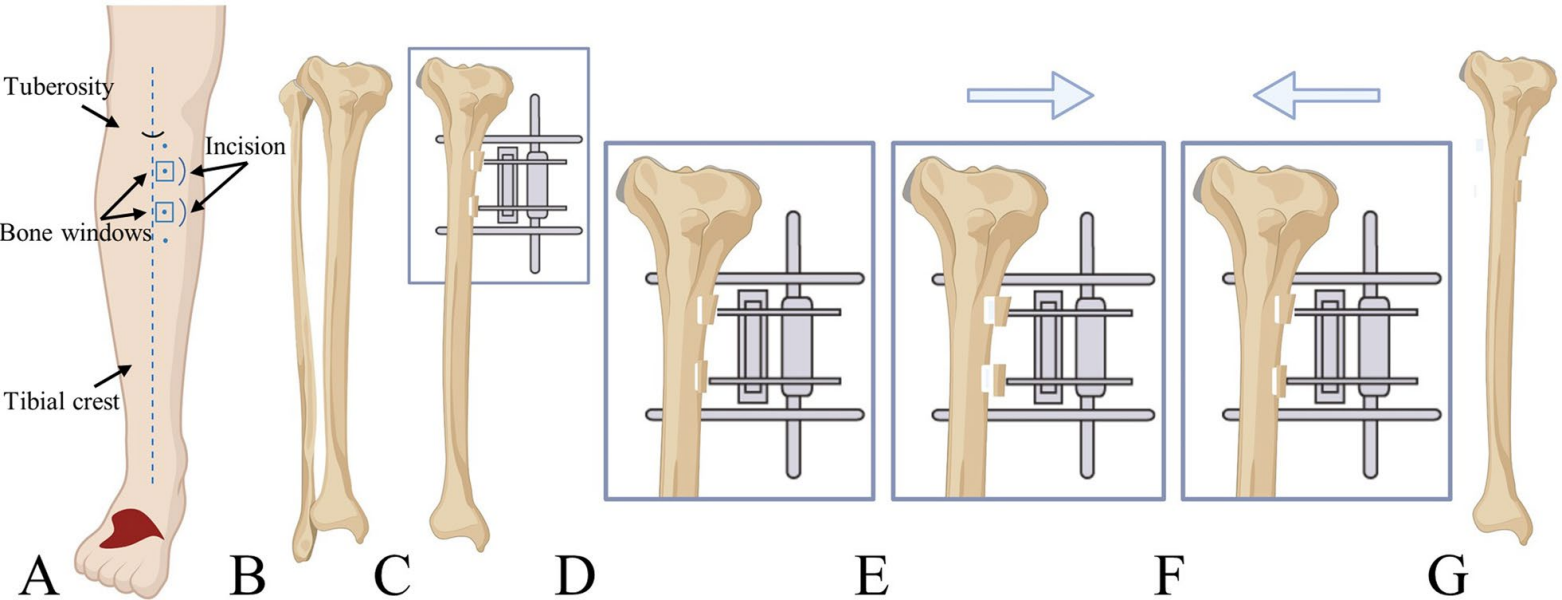
Schematic diagram of modified TTT. **A** At the medial aspect of the tibial crest, precisely 2 cm distal to the tibial tuberosity, two bone windows measuring 1.5 cm × 1.5 cm each were created with a 3-cm separation between them. **B-D** The bone flaps were meticulously detached at the designated location, followed by applying an external fixation device. **E** Starting from the 5th day after surgery, continuous traction was applied to the bone flaps. **F** After 2 weeks of traction, the direction of traction was reversed, and the bone flaps were gradually guided back to their original position. **G** The external fixation device was removed, and the tibial cortical bone gradually healed

Patients’ blood glucose levels were monitored, wound dressings were regularly changed, and the proper use of antimicrobial, anticoagulant, and anti-edema medications was ensured. Routine needle track care was performed, and alcohol was used to sterilize the external fixator. Traction was initiated on postoperative day 5. During the subsequent 14 days, the bone flaps gradually moved outward at a rate of 0.25 mm per 6 h [21] (Fig. 1). Two weeks later, radiography was performed. After maintaining this position for 3 days, the bone flaps were moved inward at the same speed. After two weeks, the bone flaps gradually returned to their original positions. Moreover, follow-up radiographic images were obtained to assess bone healing. Complete ulcer healing was considered when the wound had completely epithelialized without any drainage and remained stable for 2 weeks [22]. Once the bone healed and stabilized, the external fixation device was removed. Finally, the operated leg was protected using a brace for 6–8 weeks.

### Learning curve

Patients were numbered according to the date of surgery. The learning curve based on the operation time was calculated using the CUSUM analysis [23]. The equation is defined as: $CUSUM = \sum\nolimits_{i = 1}^{n} {\left( {yi - \overline{y}} \right)}$, where *yi* indicates the operation time for each case, $\overline{y}$ represents the average operation time for all cases, and n represents the number of consecutive cases. A scatter plot was generated using consecutive cases and CUSUM values, and the scatter plot was fitted using IBM SPSS (version 25.0; IBM Corp., Armonk, NY, USA) to obtain the functional equation. The slope of the equation was used to estimate the cut-off point of the learning curve, dividing the 60 patients into the learning and mastery groups. The cut-off point corresponded to the minimum number of cases required for a physician to accumulate experience [24]. Furthermore, it was assumed that the best-fit line in the case-time scatter plot consisted of two straight lines connected at the cut-off point. Thus, the fitting equation was defined as: *y* = a—bn (*n* ≤ n′), *y* = c (*n* > n′), where *y* represents the operation time, *n* represents the number of cases, n′ represents the cut-off point, and a, b, and c were constants. The model’s degree of fit was similarly evaluated. The fitting degree of the curve was determined by the coefficient of determination R^2^: the closer the coefficient was to 1, the higher the degree of model fitting.

### Perioperative parameters

Surgical time, intraoperative blood loss, and the number of intraoperative fluoroscopies were recorded according to the operative notes. Operative time was defined as the duration from the initial skin incision to the completion of wound closure. Intraoperative blood loss was estimated by summing the blood collected in the suction device, the amount of gauze consumed, and hidden blood loss.

### Clinical outcomes

The length of hospital stay, wound healing time, ulcer area at different time points, and complications were recorded. To evaluate the efficacy, the pain visual analog scale (VAS), ankle brachial index (ABI), and WIFI classification [25] were used as evaluation indicators. For subsequent efficacy statistics, follow-up data from patients who died during the follow-up period were excluded.

### Statistical analysis

The trial was designed by the first author. Data analysis was performed using IBM SPSS version 25.0. Data are presented as mean ± standard deviation for normally distributed continuous variables, median (interquartile range) for non-normally distributed continuous variables, and number (%) for categorical variables. The Kolmogorov–Smirnov (K-S) test was used to test the normality of the data. For continuous variables with normal distribution and homogeneity of variance, an independent sample t test was performed for group comparisons. Furthermore, the Mann–Whitney U test was used for continuous variables and ordinal data that did not follow a normal distribution. The Fisher’s exact test was used to compare dichotomous data, while for normally distributed continuous variables before and after surgery, a related sample t test was performed. Wilcoxon test was used for non-normally distributed continuous variables and ordinal data before and after surgery. Finally, ANOVA was used for comparisons between groups. Statistical significance was set at P-value < 0.05 significant.

## Results

### Study population

Based on the exclusion criteria, three patients were excluded from the study. One patient died during the follow-up period due to an accident, and two patients had incomplete follow-up data. Therefore, 60 patients were enrolled in this study. The average age of the included patients was 63.43 ± 12.52 years, with a mean diabetes duration of 20.28 ± 7.96 years. Of the participants, 39 (65%) were male, and 21 (35%) were female. Furthermore, 51 patients (85%) were unable to stabilize their blood glucose levels within the normal range. Moreover, there were 5 (8.33%), 15 (25%), 18 (30%), and 22 (36.67%) patients with ≥ 3 gangrenous toes, 2 gangrenous toes, 1 gangrenous toe, and no gangrenous toes before admission, respectively (Table 1).

**Table 1:** Patient characteristics (n = 60)

Characteristic	Learning phase (n = 20)	Mastery phase (n = 40)	*P*-value	Z/t
Age (years)	66.20 ± 11.81	62.05 ± 12.77	0.229	1.216
Male sex	12 (60)	27 (67.5)	0.579	–
The course of the disease (years)	21.35 ± 8.22	19.75 ± 7.88	0.513	− 0.654
Poor blood sugar control	18 (90)	33 (82.5)	0.704	–
Number of gangrenous toes				
≥ 3	1 (5)	4 (10)	0.15	− 1.440
2	4 (20)	11 (27.5)		
1	5 (25)	13 (32.5)		
0	10 (50)	12 (30)		
Vascular stenosis (0 as none;1 as mild; 2 as moderate; 3 as severe; 4 as occlusion)				
Anterior tibial artery	2.85 ± 1.04	2.87 ± 1.14	0.869	− 0.165
Posterior tibial artery	2.45 ± 1.19	2.55 ± 1.18	0.788	− 0.268
Peroneal artery	2.05 ± 0.76	1.63 ± 0.87	0.061	− 1.875

Data are presented as mean ± standard deviations or n (%)

### Learning curve

**Fig. 2:**
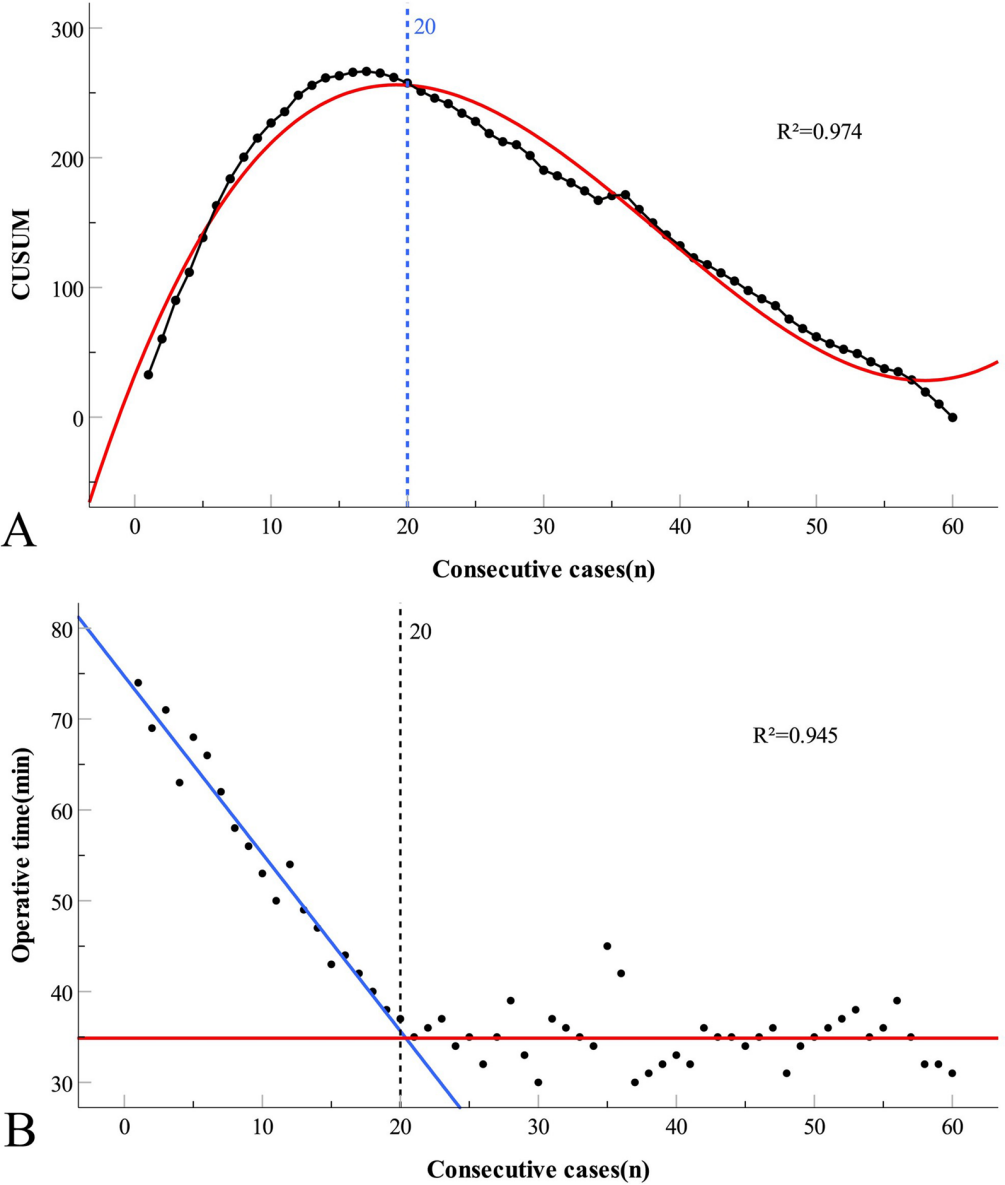
Learning curve scenarios for TTT. **A** Cumulative sum (CUSUM) plot based on operation time. The fitting curve equation is: *CUSUM* = 32.19 + 26.22 × *n*-0.91 × *n*^2^ + 7.85–3 × *n*^3^, R^2^ = 0.974.** B** Piecewise linear regression plot based on operation time. The function formula is: *y* = 74.711–1.953 × *n* (*n* ≤ 20); *y* = 34.875(*n* > 20), R^2^ = 0.945

Curve fitting was performed on the scatter plots generated based on the CUSUM values. With the accumulation of surgical cases, the CUSUM values based on operation time initially exhibited a steep increase and then steadily decreased. According to the fitting equation, when an operator completed approximately 20 operations, the slope of the curve changed from positive to negative. This indicates that the operator successfully surpassed the learning stage, and the operation time tended to stabilize **(**Fig. 2A**)**.

The case-time curve declined steeply in the early stages, and with the accumulation of surgical cases, the operation time gradually shortened and reached a plateau in the early phase. Furthermore, the case-time curve can be roughly divided into two stages. The application of a piecewise linear equation to fit the scattered data points demonstrated a good degree of fit, thereby confirming the presence of a cut-off point (Fig. 2B).

Based on CUSUM and piecewise linear regression, the cut-off point of the TTT learning curve was determined for the 20th case. The cut-off point divides the physician's learning process into two phases: the learning phase (cases 1–20, January 2020 to August 2020, lasting for 8 months) and the mastery phase (cases 21–60, September 2020 to July 2021, lasting for 11 months). The baseline data of the two groups was similar (*P* > 0.05; Table 1).

### Perioperative parameters

With an increase in proficiency, the perioperative parameters showed gradual improvement. The average operation time was significantly shortened from (54.20 ± 11.73) to (34.88 ± 3.05) minutes. Similarly, the mean number of intraoperative fluoroscopies decreased from (16.9 ± 4.66) to (9.75 ± 1.41). These improvements were statistically significant (*P* < 0.05; Table 2). However, there was no significant difference in intraoperative blood loss between the two groups (*P* > 0.05).

**Table 2:** Variables related to surgery (n = 60)

Variable	Learning phase (n = 20)	Mastery phase (n = 40)	*P*-value	Z
Operating time (min)	54.20 ± 11.73	34.88 ± 3.05	< 0.001	− 6.035
EBL (ml)	48.50 ± 7.45	46.00 ± 7.09	0.318	− 0.998
Fluoroscopy times	16.90 ± 4.66	9.75 ± 1.41	< 0.001	− 5.900

Data are presented as mean ± standard deviations

*EBL* estimated blood loss

### Clinical outcomes

**Fig. 3:**
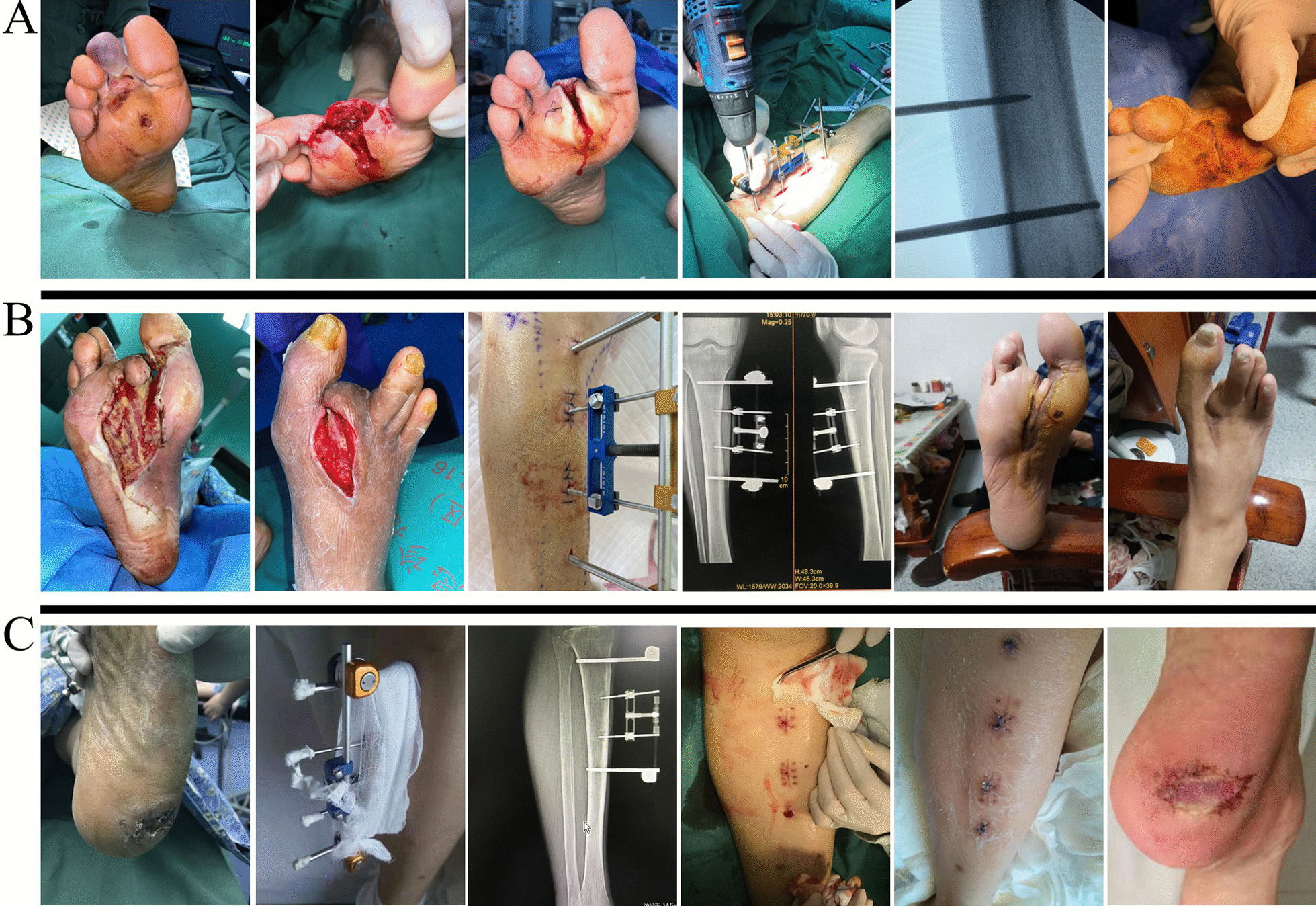
Effects of TTT on recalcitrant ulcers. **A** In this case, a 42-year-old man had a right foot ulcer lasting for 7 months, with subsequent necrosis of the second toe occurring 3 days before admission. Before the operation, the patient's right foot exhibited significant swelling, with bruised and purple skin on the second toe, and the skin on the dorsum of the toe was ulcerated with purulent exudate. The patient underwent toe amputation after admission. Furthermore, one week after the amputation, the patient underwent TTT. The external fixator was removed 1 month after the operation, and the ulcer had completely healed. **B** A 70-year-old male with a history of diabetic foot-related right second toe amputation displayed ulceration and purulence on the foot's plantar and dorsal aspects near the affected area. The patient underwent TTT followed by three subsequent debridements. Three months after TTT, the ulcers on the plantar and dorsal foot of the patient were healed. **C** A 69-year-old man presented with a 1-year ulcer of the left heel. Topical interventions were ineffective. The patient underwent TTT. The external fixator was removed 1 month after the operation. Half a month after removing the external fixator, the pinhole of the external fixation needles and the ulcer were healed

The mean hospital stay was (52.00 ± 25.74) days, and the mean time for ulcer healing was (44.83 ± 21.83) days. Herein, we present three typical cases of ulcer healing (Fig. 3). There were no significant differences in the aforementioned indicators between the groups (*P* > 0.05, Table 3).

**Table 3:** Clinical outcomes (n = 58)

Variables	Learning phase (n = 19)	Mastery phase (n = 39)	*P*-value	Z/t
Length of stay (days)	52.55 ± 23.96	51.72 ± 26.88	0.736	− 0.338
Healing time (days)	46.53 ± 21.15	44.00 ± 22.38	0.683	0.411
Incomplete healing	1 (5.3)	(5.1)	> 0.99	–
Complications	2 (10)	3 (7.5)	0.676	–

Data are presented as mean ± standard deviations or n (%)

Patients who died during the follow-up period were not included in the efficacy analysis

Complications between surgery and the last follow-up occurred in seven patients (11.7%) (Table 4). Among them, three cases (15%) occurred during the learning phase and four (10%) during the mastering phase. During the operation, three instances of drill bit fractures were observed, and all bits were successfully recovered. One of these fractures occurred during the learning phase, and the patient experienced postoperative bone necrosis. The other two fractures occurred during the mastering phase, with one patient developing bone necrosis. Moreover, one patient in the learning phase and two patients in the mastering phase underwent toe amputation due to local infection. During the study period, one patient died of myocardial infarction, while during the mastering period, one died of gastrointestinal hemorrhage. Patients who died during the follow-up period were excluded from the postoperative efficacy statistics. Overall, the incidence of complications was lower in the master’s group; however, the difference between the two groups was not statistically significant (*P* > 0.05).

**Table 4:** Details of adverse events

Adverse events	No	No. of cases occurred
Drill bit broken	3	12nd^a^, 49th^b^, 51st^b^
Osteonecrosis	2	12nd^a^, 49th^b^
Local infection	3	11st^a^, 26th^b^, 53rd^b^
Gastrointestinal bleeding	1	4th^a^
Myocardial infarction	1	36th^b^

The patient No. 51 encountered a broken drill bit; however, subsequent examination revealed no signs of osteonecrosis

^a^Learning phase

^b^Mastery phase

One patient (5.3%) in the learning stage and two patients (5.1%) in the mastering stage did not achieve complete healing, and the overall healing rate was 94.8% (3/58). After the operation, a significant reduction in the ulcer area was observed, and the VAS, ABI, and WIFI grades significantly improved (*P* < 0.05, Table 5). There was no significant difference in the efficacy evaluation indices between the two groups (*P* > 0.05).

**Table 5:** Assessment indicators (n = 58)

Variables	Learning phase (n = 19)	Mastery phase (n = 39)	*P*-value	F
Ulcer area (cm^2^)			0.262	1.287
Preoperative	15.64 ± 12.99	13.93 ± 14.90		
Last follow-up	0.16 ± 0.69	0.08 ± 0.35		
Z	− 3.517	− 5.446		
*P*-value	< 0.001	< 0.001		
VAS			0.733	0.117
Preoperative	5 (5,7)	6 (5,7)		
Last follow-up	0 (0,2)	0 (0,2)		
Z	− 3.860	− 5.476		
*P*-value	< 0.001	< 0.001		
ABI			0.27	1.243
Preoperative	0.55 ± 0.03	0.56 ± 0.03		
Last follow-up	0.66 ± 0.06	0.65 ± 0.05		
t	− 8.653	− 11.228		
*P*-value	< 0.001	< 0.001		
WIFI				
W			0.941	0.005
Preoperative	2 (2,2.75)	2 (2,2)		
Last follow-up	0 (0,0)	0 (0,0)		
Z	− 4.017	− 5.889		
*P*-value	< 0.001	< 0.001		
I			0.531	0.397
Preoperative	2 (1,2)	1 (1,2)		
Last follow-up	1 (1,1)	1 (1,1)		
Z	− 3.207	− 3.750		
*P*-value	< 0.001	< 0.001		
Fi			0.384	0.77
Preoperative	2 (2,2)	2 (2,2)		
Last follow-up	0 (0,0)	0 (0,0)		
Z	− 4.264	− 5.938		
*P*-value	< 0.001	< 0.001		

Data are presented as mean ± standard deviations or median (interquartile range)

*VAS* Visual analog scale, *ABI* Ankle brachial index, *W* wound, *I* ischemia, *Fi* foot infection

## Discussion

TTT has shown potential in stimulating microcirculation regeneration, improving the ischemic condition of the DFU and promoting healing of the ulcer wound by transverse transport of the bone flaps. Because the TTT is a relatively new surgical technique, it requires a learning and practice phase for doctors to acquire mastery. During the learning phase, several valuable yet unknown factors, including the intricacy of the surgery, safety, and actual therapeutic efficacy of this operation, need to be ascertained. Notably, the learning curve reflects the speed of skill acquisition within a certain period and serves as an assessment tool for evaluating the safety and difficulty of new technologies, minimizing unnecessary learning costs and providing references for physicians.

The most significant result of this study was the determination of the cut-off point for the learning curve of the TTT, which was calculated in the 20th case using CUSUM analysis and the piecewise linear model. The results indicated that operators could master the TTT operation after approximately 20 cases, whereas limb-lengthening surgery using Ilizarov’s law requires a longer accumulation of 60 cases for proficiency [26]. Thus, the steep learning curve of TTT suggests that this technique is relatively easy[27].

DFU falls under the category of foot and ankle surgery and can be performed by foot and ankle surgeons. Despite the differences in technology and disease, the length of the learning period can indicate the level of simplicity or complexity associated with new technologies. Therefore, we reviewed the recent foot and ankle surgery literature to describe the learning curves. Specifically, it was demonstrated that the operation time for total ankle arthroplasty tends to stabilize after accumulating 28 cases [28]. However, the study was conducted by experienced specialists, and the cut-off point may have been estimated prematurely. Notably, third-generation percutaneous chevron and akin osteotomies require physicians to perform 38–40 procedures before reaching proficiency [29, 30]. In contrast, the TTT has an earlier accessible cut-off point and is a relatively easy surgical procedure. Furthermore, the perioperative parameters of the TTT improved with the accumulation of case numbers. With an increase in the number of cases, the operation time of the operator significantly decreased and gradually stabilized, with an average reduction of 19.32 min in the mastering stage compared to the learning stage. This is consistent with the general trend observed in foot and ankle surgery [31]. In addition, this study confirmed that the number of intraoperative fluoroscopies is a factor that implies surgical proficiency. The mastering stage exhibited a substantial 6.15-fold reduction in fluoroscopy usage compared to that of the learning stage, and this disparity held statistical significance. This difference can be attributed to the operator's growing familiarity with anatomy and key surgical procedures. Although intraoperative blood loss was reduced during the mastering phase, the difference was not statistically significant. The relatively lower blood loss in TTT procedures, combined with the greater impact of changes in operation time on overall blood loss, suggests that intraoperative blood loss may not be suitable for assessing the learning curve of TTT. With gradual improvement in self-confidence, surgeons may try to deal with relatively difficult cases and adjust the operation steps, resulting in prolonged operation times and extreme values that partly deviate from the curve, causing the curve to fluctuate.

Here, the area of the bone window was further reduced based on the original surgery, and satisfactory results were obtained. The overall ulcer healing rate was 94.8%, and the efficacy evaluation indices (VAS, ABI, and WIFI grades) showed significant improvement compared with their preoperative values, similar to traditional surgical procedures [32, 33]. However, whether different bone window sizes affect the stimulatory effect of the subsequent distraction on the periosteum needs to be proven through rigorously controlled experiments. The ulcer healing rate with traditional treatment modalities (including surgical debridement, free-flap transplantation, infection control, and revascularization) ranges from 56–77% [34, 35]. Moreover, TTT results in a higher rate of ulcer healing and has obvious advantages in treating DFU, particularly intractable DFU. In general, owing to their lack of experience, surgeons may not be able to achieve the expected results in the learning phase. However, our study found no significant differences in the efficacy evaluation indicators between the two groups. This indicates that the TTT is a relatively safe surgical procedure that can yield stable clinical outcomes, even in the early stages of the learning process. As wound healing time was primarily dependent on the initial ulcer area of the included patients, there was no significant difference in healing time between the two groups. Additionally, the surgical effect depends on the physician’s understanding of the indications. The TTT may not yield favorable therapeutic results in patients with severe occlusion of large vessels in the lower extremities, and caution should be exercised when considering the TTT in such patients. Currently, the surgical indications for TTT require further refinement.

Reducing the osteotomy area has several advantages in TTT surgery. First, it helps avoid extensive stripping of the periosteum, which preserves the blood supply to the bone and minimizes necrosis of the free bone blocks. Second, it can maximize the protection of the stability and biomechanical integrity of the tibia. Specifically, the secondary tibial fracture rate associated with traditional large-scale osteotomy has been reported to be 2% [36], whereas no tibial fractures were observed in this study. This indicates that reducing the area of the bone window is a key factor in avoiding tibial fractures. Additionally, the smaller osteotomy area allowed patients to move out of bed as soon as possible, reducing the risk of lower-extremity vein thrombosis.

The total complication rate observed in this study was 11.67% (7/60), which falls within the range of rates (5.26–20.9%) reported in other studies [20, 32, 33, 37]. Moreover, the incidence of complications during the learning phase was slightly higher than that during the mastery phase; however, the difference was not statistically significant. Thus, these findings imply that surgeons interested in this procedure can be reassured that manipulation during the learning phase is unlikely to cause additional harm to the patients. Nonetheless, the lower incidence of complications in the mastery phase suggests that with increasing familiarity, surgeons can minimize these potential complications to a certain extent. Therefore, ensuring that beginners receive adequate supervision from experienced surgeons during the first 20 surgical procedures is crucial.

The surgical procedure for TTT is not complicated; therefore, surgeons can quickly master TTT after systematic study and training. However, an irregular operation affects treatment efficacy and increases the incidence of complications. Based on our clinical experience and relevant literature, the precautions for TTT surgery are summarized as follows. (1) Selection of surgical incision: The lateral tibia is close to the common peroneal nerve; thus, the operation on the medial tibia can avoid injury to the common peroneal nerve. (2) Selection of bone windows: The distal 1/3 of the tibia has poor blood supply and is relatively thin, making it prone to fractures. It is recommended that the starting point of the incision should be 2 cm below the tibial tuberosity. (3) Osteotomy method: Directing the bone drill perpendicular to the bone surface can minimize damage to the periosteum, and using a bone chisel for osteotomy can reduce thermal damage to the bone tissue caused by an electric saw. (4) Bone transport time: The medial tibia has less soft tissue and poorer blood supply. Immediate transport after surgery can create excessive tension, which is unfavorable for healing. Delayed transport, initiated 5 days postoperatively, can effectively reduce bleeding and alleviate early postoperative pain [38]. (5) Transport method: Implementing reverse transport helps maintain the position of the bone blocks and prevents their uplift of the bone blocks. Furthermore, it prolongs the duration of microcirculatory regeneration stimulated by traction. (6) Skin care: Interrupted sutures are advised when closing an incision. If the skin is in close proximity to the external fixator, the incision should be extended appropriately to reduce tension and prevent eversion. To avoid excessive tension caused by bone segment movement and to prevent skin necrosis in the osteotomy area, it is important to monitor the skin's condition around the incision site. Moreover, it is important to take precautions to protect the contralateral limb and prevent the sharp edges of the external fixator from injuring the skin on the opposite side. (7) Moving speed and frequency: The optimal moving speed for TTT is 1 mm/d, which can achieve a favorable tissue regeneration effect. Furthermore, high-frequency stimulation imposes less microtrauma on the tissue and extends the duration of the mechanical microenvironment around the tissue [39]. High-frequency traction upregulates the expression of angiogenic mediators and promotes the formation of new blood vessels [40]. Hence, transport should be performed four times daily rather than once.

One way to quickly accumulate surgical experience is to perform intensive training on the key steps of the TTT, such as tibial fenestration and pin placement. Moreover, physicians can enhance their skills using cadavers or anatomical models. Additionally, it is necessary to realize that DFU results from the confluence of multiple factors. Therefore, it is worth paying attention to professional wound care, anti-infection treatments, and strict control of blood glucose levels during treatment are important to prevent the occurrence of cardiovascular and cerebrovascular complications.

## Conclusions and limitations

Our study has some limitations. First, the operations were performed by the same surgeon, which introduces certain subjective factors. Simultaneously, objective factors such as the number of patients, medical insurance policies, and different devices all play an important role in the learning process. Therefore, other surgeons may have used cut-off points earlier or later than we did. Second, the operation time is a key factor in determining whether the surgeon has overcome the learning curve, and TTT has fewer complications, which has a weak predictive effect on the learning curve; therefore, we did not include complications and failed operations in our analysis. Third, this study included only patients with DFU whose lower-extremity vessels were not completely blocked. However, the repair function of TTT in large and medium vessels requires further investigation. Lastly, the short duration of follow-up and the retrospective design are limitations of this study.

Nevertheless, scientific statistical methods and appropriate methods for describing the learning curve ensured the credibility of the findings of this study. TTT is a novel surgical procedure that is relatively simple to master, can effectively treat refractory DFU, and can serve as a viable alternative treatment for DFU. This study provides technical references and experiences with TTT, thereby reducing unnecessary learning costs and providing guidance for organizations or individuals seeking to enhance their TTT proficiency. In the future, it will be imperative to conduct research on the learning curves of beginners at multiple centers to mitigate the interference of subjective factors.

## Data Availability

The datasets collected during and/or analyzed during the current study are available from the corresponding author upon reasonable request.
